# Characterization of CM-398, a Novel Selective Sigma-2 Receptor Ligand, as a Potential Therapeutic for Neuropathic Pain

**DOI:** 10.3390/molecules27113617

**Published:** 2022-06-04

**Authors:** Lisa L. Wilson, Amy R. Alleyne, Shainnel O. Eans, Thomas J. Cirino, Heather M. Stacy, Marco Mottinelli, Sebastiano Intagliata, Christopher R. McCurdy, Jay P. McLaughlin

**Affiliations:** 1 Department of Pharmacodynamics, College of Pharmacy, University of Florida, Gainesville, FL 32610, USA; lisawilson@ufl.edu (L.L.W.); amyalleyne@cop.ufl.edu (A.R.A.); shaieans@cop.ufl.edu (S.O.E.); thomas.cirino@ucsf.edu (T.J.C.); heather.stacy12@gmail.com (H.M.S.); 2 Department of Medicinal Chemistry, College of Pharmacy, University of Florida, Gainesville, FL 32610, USA; m.mottinelli@cop.ufl.edu (M.M.); s.intagliata@unict.it (S.I.); cmccurdy@cop.ufl.edu (C.R.M.)

**Keywords:** sigma, sigma-2 receptor, sigma-2 ligand, allodynia, analgesia, neuropathic pain, sedation, addiction

## Abstract

Sigma receptors modulate nociception, offering a potential therapeutic target to treat pain, but relatively little is known regarding the role of sigma-2 receptors (S2R) in nociception. The purpose of this study was to investigate the in vivo analgesic and anti-allodynic activity and liabilities of a novel S2R selective ligand, 1-[4-(6,7-dimethoxy-1,2,3,4-tetrahydroisoquinolin-2-yl)butyl]-3-methyl-1,3-dihydro-1,3-benzimidazol-2-one (CM-398). The inhibition of thermal, induced chemical, or inflammatory pain as well as the allodynia resulting from chronic nerve constriction injury (CCI) model of neuropathic pain were assessed in male mice. CM-398 dose-dependently (10–45 mg/kg i.p.) reduced mechanical allodynia in the CCI neuropathic pain model, equivalent at the higher dose to the effect of the control analgesic gabapentin (50 mg/kg i.p.). Likewise, pretreatment (i.p.) with CM-398 dose-dependently produced antinociception in the acetic acid writhing test (ED_50_ (and 95% C.I.) = 14.7 (10.6–20) mg/kg, i.p.) and the formalin assay (ED_50_ (and 95% C.I.) = 0.86 (0.44–1.81) mg/kg, i.p.) but was without effect in the 55 °C warm-water tail-withdrawal assay. A high dose of CM-398 (45 mg/kg, i.p.) exhibited modest locomotor impairment in a rotarod assay and conditioned place aversion, potentially complicating the interpretation of nociceptive testing. However, in an operant pain model resistant to these confounds, mice experiencing CCI and treated with CM-398 demonstrated robust conditioned place preference. Overall, these results demonstrate the S2R selective antagonist CM-398 produces antinociception and anti-allodynia with fewer liabilities than established therapeutics, adding to emerging data suggesting possible mediation of nociception by S2R, and the development of S2R ligands as potential treatments for chronic pain.

## 1. Introduction

Over half the people experiencing chronic pain in the United States respond poorly to current treatments [1]. Approved therapeutics for chronic pain presently include repurposed antidepressants such as tricyclic antidepressants (TCAs), antiseizure medications such as gabapentin, and opioids [2], but these exhibit major liabilities such as sedation, increased fall-risk, tolerance, addiction, and other psychoactive effects [3,4]. This points to the need for more effective and safer therapeutics for chronic pain.

The sigma receptors are ligand-operated transmembrane chaperone proteins that are expressed throughout the central and peripheral nervous systems [5,6,7]. These receptors are classified into two distinct receptor subtypes: sigma-1 and sigma-2 [5,6,7]. Cloned in 1996 [8], sigma-1 receptor (S1R) modulation has shown therapeutic promise for alleviating chronic pain [9,10]. The role of the sigma-2 receptor in nociception is less clear. First identified as Tmem97, the sigma-2 receptor (S2R) was only recently cloned in 2017 [11] and is known to regulate intracellular calcium and cholesterol homeostasis [12,13], but insights into the functional role of this receptor in physiological and pathological conditions remain limited, hampered by a paucity of sigma-2 receptor selective ligands. Research conducted with existing sigma-2 ligands has focused on their potential to treat cancer [14,15], although CT1812 is currently in phase 1 clinical trials for the modulation of neurodegenerative diseases such as Alzheimer’s [16]. Extending this, emerging studies with new S2R-selective ligands have suggested they, too, may modulate nociception.

**Figure 1:**
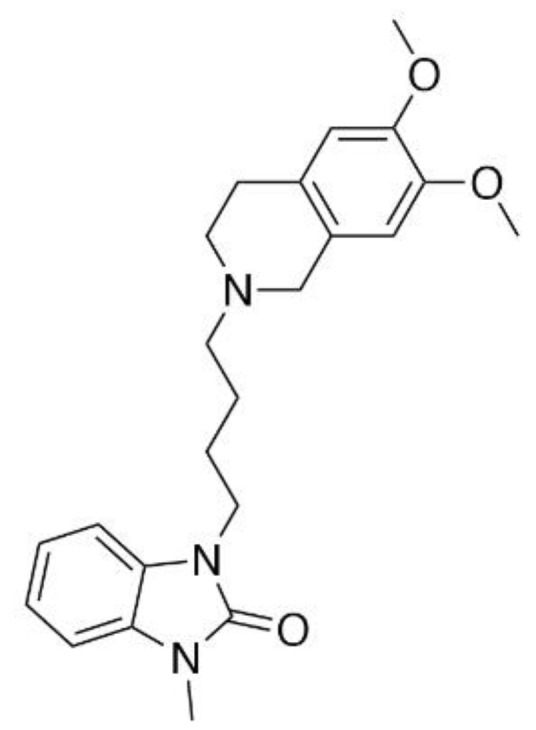
Chemical structure of 1-[4-(6,7-dimethoxy-1,2,3,4-tetrahydroisoquinolin-2-yl)butyl]-3-methyl-1,3-dihydro-1,3-benzimidazol-2-one (CM-398).

CM-398 (Figure 1) is an analog of the established sigma-1 antagonist CM-304 [9,17], synthesized in an attempt to increase the duration of the pharmacological action of the parent compound. With a *K_i_* value of 0.43 ± 0.015 nM for sigma-2 receptors, Intagliata and colleagues [17] reported that CM-398 is more than 1000-fold more selective for the sigma-2 over the sigma-1 receptor, providing a useful S2R-selective ligand with which to probe the involvement of sigma-2 receptors in nociception. It is not conclusively known if CM-398 acts as a sigma-2 receptor agonist or antagonist. In preliminary testing, CM-398 was shown to ameliorate inflammatory pain produced by the formalin assay [17], but a full antinociceptive and anti-allodynic evaluation as well as potential clinical liabilities were not assessed. Accordingly, we presently evaluated CM-398 for its ability to modulate multiple modalities of acute and chronic pain in both reflexive and operant models, and assessed liabilities of sedation, respiratory depression, and abuse potential in a place preference assay.

## 2. Results

### 2.1. Assessment of CM-398 Antinociception in Visceral, Inflammatory, and Acute Thermal Models of Nociception

**Figure 2:**
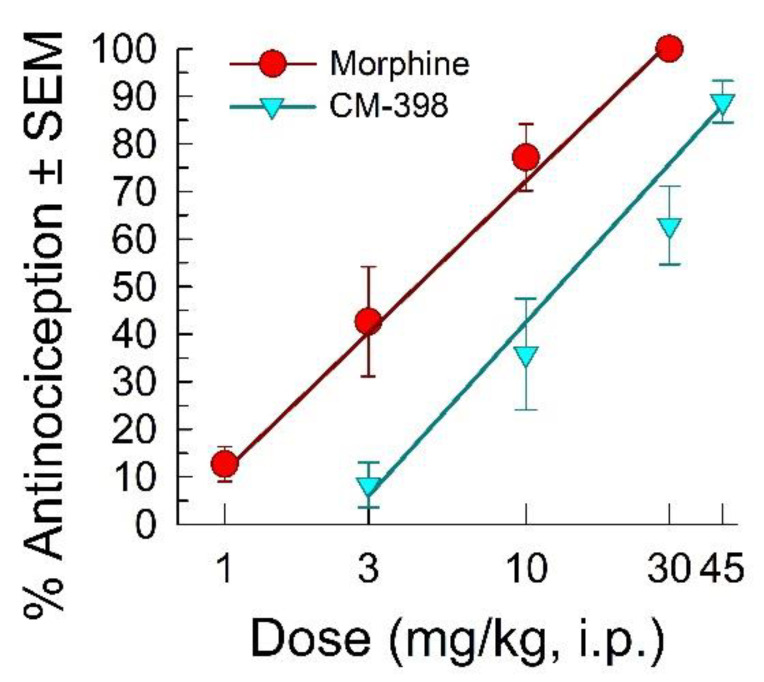
Dose-dependent antinociception of sigma-2 receptor ligand CM-398 following i.p. administration in the mouse acetic acid writhing test. Opioid agonist morphine is shown as a positive control. All points represent average response ± SEM at peak effect, 30 min after admin in 8–10 mice. ED_50_ values analyzed using linear regression.

**Figure 3:**
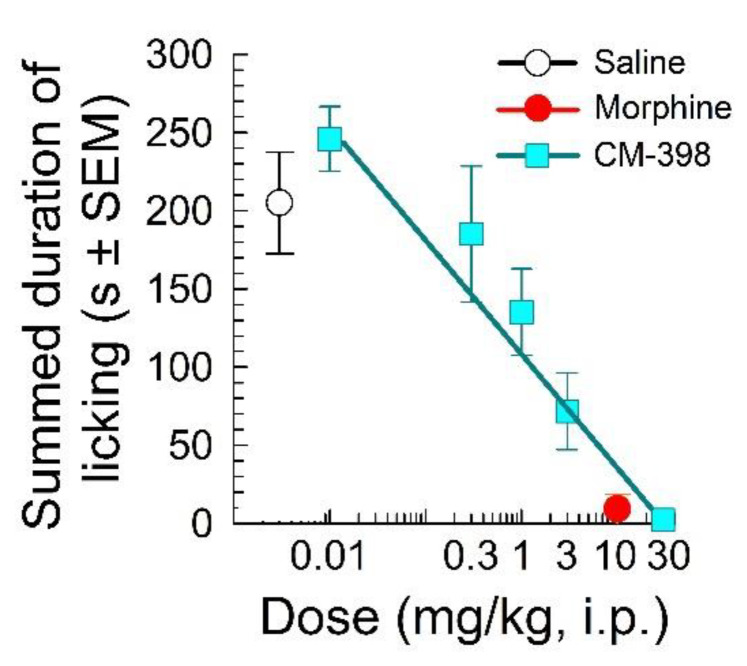
Evaluation of CM-398 for antinociceptive effects in the mouse formalin-induced inflammation assay. Dose-dependent antinociception of sigma-2 receptor ligand CM-398 followed i.p. administration. Control mice were treated with saline (0.9%, i.p.) or morphine (10 mg/kg, i.p.). All points represent summed time spent licking ± SEM administered to 5–10 mice for all points.

CM-398 dose-dependently attenuated nociception in the acetic acid writhing test with an ED_50_ (and 95% C.I.) value of 14.7 (10.6–20) mg/kg, i.p. (Figure 2). The antinociception was less potent than the effects of both the parent compound CM-304 (reported as 0.48 (0.09–1.83) mg/kg, i.p.; [9]) and morphine (with an ED_50_ and 95% C.I. value of 3.91 (1.45–10.4) mg/kg, i.p.; Figure 2). CM-398 also demonstrated significant dose-dependent antinociception after i.p. administration in the formalin-induced inflammation assay (F_(5,36)_ = 9.55, *p* < 0.001; one-way ANOVA; Figure 3), with an ED_50_ (and 95% C.I.) value of 0.86 (0.44–1.81) mg/kg. Mice spent significantly less time licking the formalin-injected paw compared to the saline control after treatment with CM-398 with doses of 3 or 30 mg/kg i.p. (*p ≤* 0.005 or better, Dunnett’s test) or morphine (10 mg/kg i.p.; *p* < 0.001; Student’s t-test vs. saline control).

**Figure 4:**
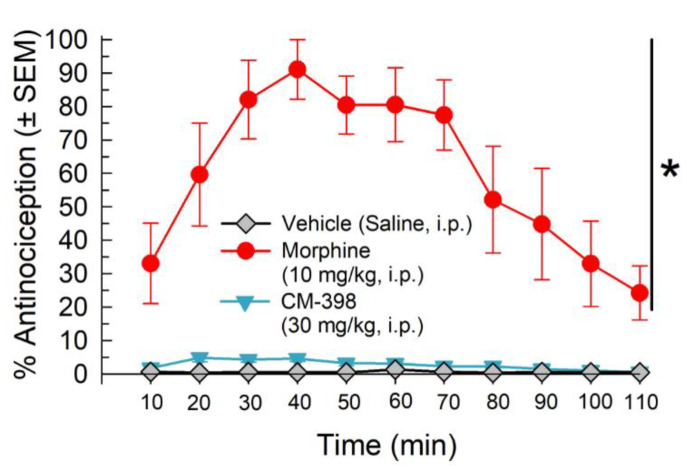
Evaluation of CM-398 for acute thermal antinociception in the 55 °C warm water tail-withdrawal assay. Mean ± SEM of latency to withdraw the tail from warm water after treatment with morphine (10 mg/kg, i.p.; red circles), CM-398 (30 mg/kg, i.p.; cyan triangles), or vehicle (0.9% saline, i.p.; gray diamonds) was examined every 10 min up to 110 min.; n = 7–8 for all points, * *p* < 0.05; two-way RM ANOVA with Tukey’s post hoc test.

In contrast, CM-398 did not produce significant antinociception in the 55 °C warm-water tail-withdrawal assay. Whereas morphine (10 mg/kg, i.p.) produced significant antinociception over time (factor: treatment: F_(2,249)_= 247.9, *p* < 0.0001; repeated measures two-way ANOVA with Tukey’s test; Figure 4), pretreatment with a 30 mg/kg, i.p. dose of CM-398 produced results that were not significantly different from the vehicle control (*p*= 0.7593; Figure 4).

### 2.2. Anti-Allodynic Effects of CM-398 in the CCI Neuropathic Pain Model

**Figure 5:**
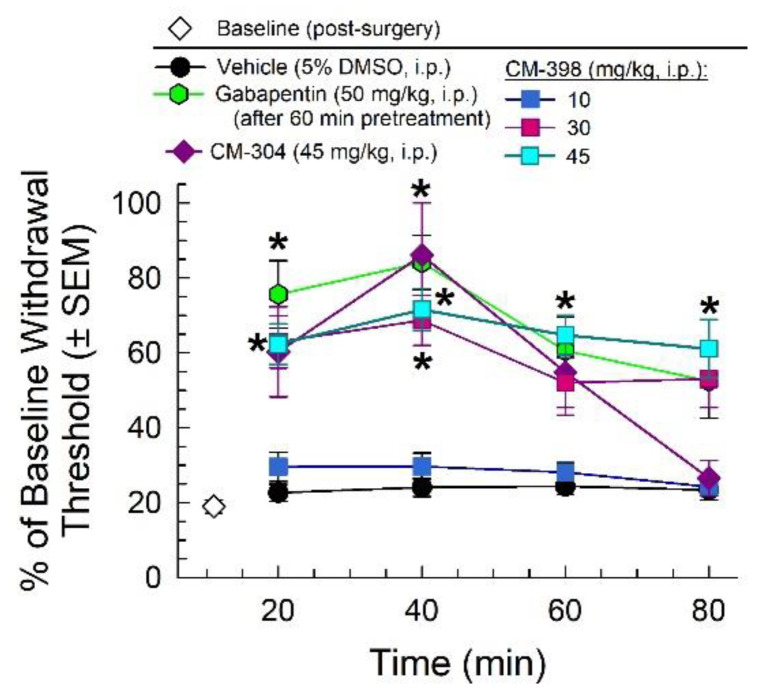
Dose- and time-dependent anti-allodynic activity of CM-398 (squares) in the mouse chronic constriction injury (CCI) model of neuropathic pain. Mechanical allodynia produced from sciatic nerve ligation was reduced from 40–80 min post-CM-398 (30 mg/kg, i.p., pink squares, and 45 mg/kg, i.p., cyan squares) in a manner similar to the positive control, gabapentin (50 mg/kg, i.p., green hexagons). CM-398 produced effects that were longer lasting than the parent compound CM-304 (45 mg/kg, i.p., dark-purple diamonds). N = 10 for all groups; * = significantly different from vehicle controls; *p* < 0.05; two-way ANOVA with Tukey post hoc test. (Note: CM-304 data were previously published in Cirino et al., 2019 [9]).

In the chronic constriction nerve injury assay, CM-398 (Figure 5) attenuated the reduced paw withdrawal threshold characteristic of mechanical allodynia caused by CCI (white diamond; Figure 5) in a significant time- and dose-dependent manner when compared to the saline control (factor: treatment: F_(5,231)_= 34.98, *p* < 0.001; two-way ANOVA with Tukey’s multiple comparisons post hoc test; Figure 5). The anti-allodynic effects of CM-398 at doses of 30 or 45 mg/kg, i.p. were equivalent to those of the positive control gabapentin (*p* > 0.5 and *p* > 0.7, respectively, for all time points). Although also equivalent to responses of the parent compound CM-304, the anti-allodynic effects of CM-398 were longer lasting, with the loss of significant CM-304 effects by 60 min, while the responses of CM-398 still significantly differed from the vehicle control 80 min after administration of either 30 mg/kg (*p* < 0.03) or 45 mg/kg (*p* < 0.0001) doses (Figure 5).

### 2.3. Assessment of CM-398-Induced Liabilities of Sedation, Respiratory Depression, and Drug-Seeking Behavior

**Figure 6:**
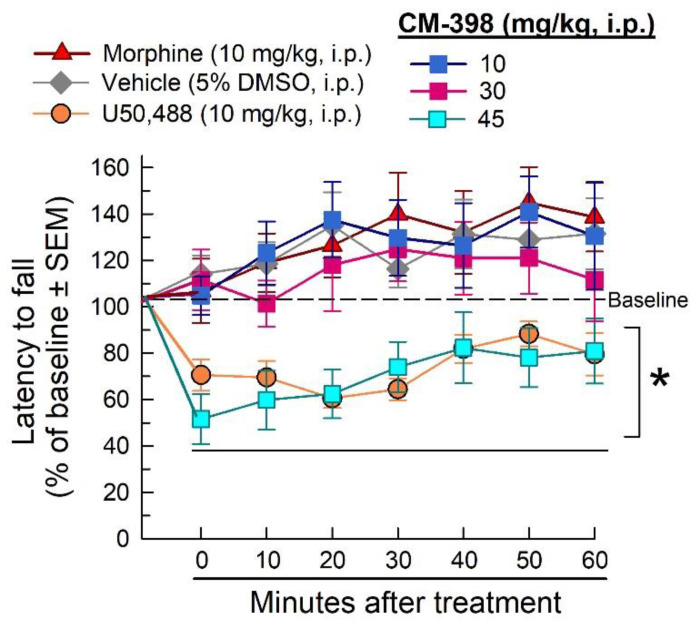
Assessment of CM-398 for dose- and time-dependent changes in evoked locomotor activity in the mouse rotarod assay. CM-398 (squares) was administered at 10, 30, or 45 mg/kg, i.p. doses prior to testing. U50,488 (10 mg/kg, i.p; orange circles) served as a positive control; * = significantly different from vehicle response (5% DMSO, i.p.; gray diamonds), *p* < 0.05; two-way ANOVA with Dunnett’s post hoc test; n = 8–12 mice/treatment.

Potential sedation and impairment of evoked, coordinated locomotion by CM-398 were evaluated in mice with the rotarod assay. As expected, morphine was without effect, whereas U50,488 (the kappa opioid receptor agonist) significantly impaired locomotion compared to vehicle (factor: treatment: F_(5,364)_ = 31.9, *p* < 0.0001, and factor: time: F_(6,364)_ = 3.2, *p* = 0.0047, two-way ANOVA with Dunnett’s post hoc test; Figure 6). While lower doses (10 and 30 mg/kg, i.p.) of CM-398 produced no sedative effects compared to the vehicle control, the high dose tested (45 mg/kg, i.p.) produced a significant impairment of evoked locomotor activity (*p* < 0.0001; Figure 6, cyan squares).

**Figure 7:**
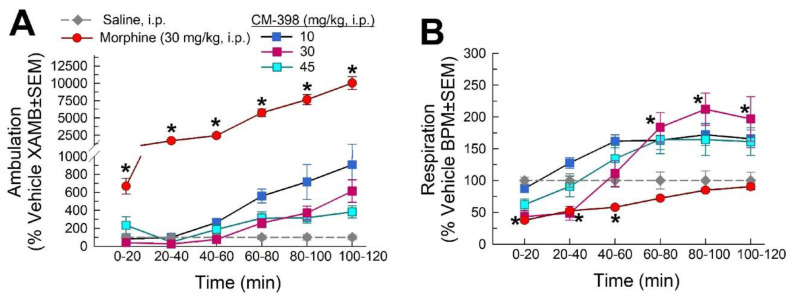
Evaluation of potential effects of CM-398 on (**A**) spontaneous ambulation and (**B**) respiration in C57BL/6J mice. Ambulation and respiration were monitored after i.p. administration of CM-398 (10, 30, or 45 mg/kg; squares), saline (grey diamonds), or morphine (30 mg/kg, red circles) using the CLAMS/Oxymax system. Data are presented as % vehicle response ± SEM for ambulation (XAMB, (**A**)), or breaths per minute (BPM, (**B**)); * = significantly different from baseline response (*p* < 0.05); n = 12 mice/treatment.

The effects of CM-398 on spontaneous locomotor activity and respiration were characterized in the Comprehensive Lab Animal Monitoring System (CLAMS). As expected, morphine (30 mg/kg, i.p.) demonstrated significant increases in ambulation across all time points (factor: treatment × time: F_(20,330)_ = 38.0; *p* < 0.0001; repeated measures two-way ANOVA with Dunnett’s post hoc test; Figure 7A). While trending towards an increase, the variable response with CM-398 did not significantly alter ambulation at any dose tested. In contrast, CM-398 produced a significant, dose-dependent, transient reduction in respiration rates up to 40 min after administration of a 30 (but not 10 or 45) mg/kg i.p. dose that was comparable to morphine-induced respiratory depression (factor: treatment x time: F_(20,330)_ = 3.07; *p* < 0.0001; repeated measures two-way ANOVA with Dunnett’s post hoc test; Figure 7B). These respiratory effects were reversed between 60 and 120 min, where higher doses of CM-398 (30 or 45 mg/kg i.p.) produced a significant increase in respiration (*p* < 0.0001). Consistent with earlier reports [9], morphine (30 mg/kg i.p.) significantly reduced respiration rates for an hour (*p* < 0.02).

**Figure 8:**
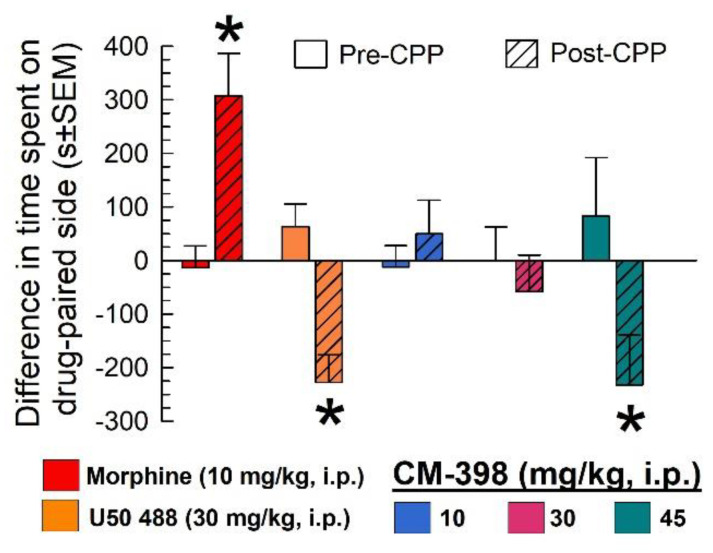
Evaluation of CM-398 in the conditioned place preference assay. While mice showed preference for the chamber paired with morphine treatment (10 mg/kg, i.p.), and aversion for the kappa agonist, U50,488 (30 mg/kg, i.p.), no preference or aversion was seen for CM-398 at 10 and 30 mg/kg, i.p. However, CM-398 at 45 mg/kg, i.p. showed significant conditioned place aversion. For each group, n = 14–24; * = postconditioning response (striped bars) significantly different from matching pre-CPP response (matching open bars), *p* < 0.05; two-way ANOVA with Sidak’s post hoc test.

Additional mice were place-conditioned for 40 min each of two days with morphine, U50,488, or CM-398 (at 10, 30, or 45 mg/kg, i.p.). As expected, morphine produced significant conditioned place preference (CPP) and U50,488 produced conditioned place aversion (CPA) (factor: treatment x conditioning: F_(4,190)_ = 2.96; *p* = 0.02; two-way ANOVA with Sidak’s post hoc test; Figure 8). In contrast, while low and medium doses of CM-398 did not show significant place preference or aversion after place conditioning with the final preference results statistically equivalent to the preconditioning responses (*p* = 0.97 each), the higher dose of CM-398 produced a significant CPA (*p* = 0.008; rightmost bars, Figure 8).

### 2.4. Assessment of CM-398 Antinociception in an Operant Pain Model

**Figure 9:**
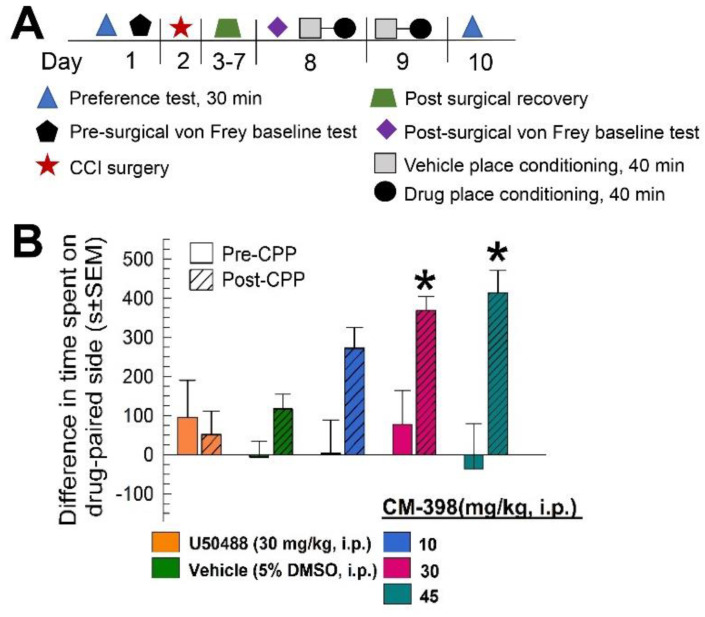
Evaluation of CM-398 in an operant model of antinociception using constrictive nerve injury (CCI)-conditioned place preference (CPP). (**A**) Schematic representation of the CCI/CPP operant model of pain protocol; (**B**) Dose-dependent antinociception of CM-398 following i.p. administration in the mouse CCI/CPP operant pain model. Negative control mice were treated with vehicle (5% DMSO, i.p.; second pair of bars from left) and positive control mice were treated with the kappa opioid agonist U50,488 (leftmost pair of bars). All points represent differences in time spent on the drug-paired side ± SEM tested in 15–20 mice/drug. * = postconditioning response (striped bars) significantly different from matching pre-CPP response (matching open bars), *p* < 0.05; two-way ANOVA with Sidak’s post hoc test.

The locomotor impairment demonstrated by high doses of CM-398 raised concern that the anti-allodynic effects observed may have been a false positive produced by immobility. To control for this, CM-398 was characterized in an operant pain model that combined the constrictive nerve injury (CCI) and conditioned place preference (CPP) assays (see schematic outline of testing; Figure 9A). In this model, mice subjected to CCI were then place-conditioned with the kappa opioid receptor agonist U50,488 (30 mg/kg, i.p.) or sigma-2 receptor ligand CM-398 (10, 30, or 45 mg/kg, i.p.) for two consecutive days. On the final day, place preference was assessed when the subjects were allowed to roam freely in the CPP apparatus in the absence of a drug. In contrast to the results with naïve mice, CCI-exposed mice place-conditioned with the selective sigma-2 receptor ligand CM-398 demonstrated significant dose-dependent conditioned place preference (factor: pre/post difference: F_(1,168)_ = 11.48, *p* < 0.0009; factor: treatment: F_(4,168)_ = 2.02, *p* < 0.094; two-way ANOVA and Sidak’s post hoc test; Figure 9B). Place conditioning with the 10 mg/kg dose of CM-398 did not result in significant differences from preconditioning place preferences (*p* = 0.26), while the 30 and 45 mg/kg, i.p. doses each resulted in significant place preference (of *p* < 0.05 and *p* < 0.0005, respectively; Figure 9B). The control CCI mice place conditioned with the vehicle aloneshowed no significant change in pre- versus post-conditioning place preference (*p* > 0.99). However, of interest, place conditioning with U50,488 (30 mg/kg, i.p.) here resulted in no significant change in preference in treated CCI mice (*p* = 0.99; Figure 9B, leftmost bars), in contrast to the result with naïve mice (Figure 8). Collectively, as the place-preference responses were assessed in the absence of any drug (i.e., a day after the last exposure), the results of this operant testing suggest the antinociceptive properties of CM-398 were independent of any locomotor effect.

## 3. Discussion

The current data found the selective sigma-2 receptor ligand CM-398 demonstrated antinociceptive and anti-allodynic activity over a variety of acute and chronic pain modalities, albeit with no efficacy in an acute reflexive model of thermal pain. Notably, despite some sedative properties at higher doses, CM-398 demonstrated reduced respiratory depression and no liabilities of conditioned place preference associated with clinically used opioids. Moreover, antinociceptive efficacy of CM-398 was confirmed free of confounding locomotor impairment with testing in an operant pain model using mice exposed to CCI in a conditioned place preference assay. Collectively, these results suggest further development of CM-398 may prove useful to individuals suffering from chronic pain conditions, while also contributing insights into the function of sigma-2 receptors.

It is appropriate to acknowledge several important caveats regarding the current results. Based on structural similarities to the parent compound and sigma-1 receptor antagonist CM-304, it was predicted that CM-398 would possess sigma receptor antagonism [17]. However, the unusual pharmacology of the sigma receptors has hampered the adaptation of conventional in vitro high-throughput screening assays, precluding direct evidence of this activity. The present state-of-the-art includes the phenytoin assay, where this antiseizure drug is interpreted to allosterically modulate the activity of the sigma-1 receptor [18,19]. In guinea pig brain membranes, coincubation with phenytoin was used to reportedly increase the binding affinity of putative sigma-1 receptor agonists while slightly decreasing the affinity of putative sigma-1 receptor antagonists [18]. In contrast, reliable functional in vitro assays for sigma-2 receptor ligands are still in development [20], potentially one day resolving this knowledge gap, but leaving confirmation of the function of this selective sigma-2 receptor ligand currently out of reach. This question is important, as some recent reports suggest that sigma-2 receptor activation may produce analgesia. UKH-1114 is a sigma-2 receptor agonist, as determined by in vivo characterization [21]. Sahn et al. reported that UKH-1114 produced dose-dependent antinociception in the spared nerve injury assay in mice [21], suggesting that the anti-allodynic activity of CM-398 herein might be attributed to sigma-2 receptor agonism. Consistent with this are recent data finding that the activation of sigma-2 receptors accentuates mu-opioid receptor agonist-mediated antinociception [22], further suggesting an analgesic effect of sigma-2 receptor agonists. Although compounds CT0109 and CT1812 are established sigma-2 receptor antagonists shown to improve cognitive performance in mouse models of Alzheimer disease [16,23], neither has been examined for analgesic or anti-allodynic properties, which might resolve this question.

A new series of novel ligands with high affinity for the sigma-2 receptor were recently shown to produce anti-allodynic effects up to 24 h [24], but the function of these compounds remains (understandably) undetermined. Additional future studies with established sigma-2 receptor ligands of known function and sigma-2 receptor knockout mice would also be beneficial to investigating this question. However, the majority of the current literature holds that antinociception and/or anti-allodynia against inflammatory and neuropathic pain states result from sigma receptor antagonism [10,25,26]. Extending this, our selective sigma-2 ligand CM-398 was found efficacious against the affective component of pain in mice subjected to CCI in an operant model. Additionally, consistent with current investigations of sigma receptor antagonists, CM-398 was unable to attenuate acute nociception induced in the 55 °C warm-water tail-withdrawal assay [27]. However, our results were able to confirm that CM-398 is more potent than morphine in the acetic acid writhing assay and equipotent with morphine in formalin tests, both of which are characterized by an increased inflammatory response in rodents [28]. Although Intagliata and colleagues reported CM-398 has selectivity for sigma-2 over sigma-1 receptors [17], it remains unclear if the potential anti-inflammatory effects of CM-398 are due to interactions with the sigma-2 or sigma-1 receptor or both. Intagliata and colleagues also reported CM-398 bound to serotonin transporters (SERT), a neurotransmitter associated with the descending control of pain [29], but the 568-fold lower affinity of CM-398 for SERT over sigma-2 receptor [17] limits the plausibility of this as a mechanism mediating the antinociception observed presently. Alternatively, it is further possible that as with sigma-1 receptor ligands, sigma-2 receptor ligands may modulate a variety of intracellular signaling inflammatory mediators such as the release of nitric oxide or bradykinin-induced calcium release at the inflamed site [30], or modulate inflammatory activity by attenuating pERK1/2 in the dorsal horn and the dorsal root ganglion [31], thereby producing analgesia. While direct examination of these nociceptive mechanisms was beyond the scope of this initial characterization study, we anticipate the establishment of selective sigma receptor antagonists will facilitate studies of the signal transduction modulated by CM-398 through actions at the sigma-1 and sigma-2 receptors to resolve these questions.

Chronic constrictive nerve injury (CCI) is a common and well-validated rodent model for neuropathic (sciatica) pain. In the clinic, neuropathic pain such as sciatica is treated with anticonvulsants such as gabapentin or opioids [2]. Similar to previously collected data with sigma receptor antagonists [9], CM-398 dose-dependently demonstrated efficacy against CCI-induced neuropathy after a single dose for a duration of time that was comparable to gabapentin and longer than its parent compound, CM-304. Peripheral nerve injury, such as CCI, produces alterations to nerve conduction in both the ascending and descending pain pathways thought to promote peripheral sensitization [32]. CCI is known to initiate the activation of TRPV1 and sodium channels on the injured peripheral nerve, increasing neuronal excitability in a manner associated with the increased perception of noxious stimuli [32]. The enhanced NMDA receptor activity and pain sensitization associated with peripheral nerve injury are blocked by the application of sigma-1 receptor antagonists [33,34]. The present anti-allodynic effects of CM-398 could also be attributed to an antagonism of sigma-2 receptors to prevent NMDA-mediated sensitization, but this has not been directly examined with sigma-2 receptors and is beyond the scope of this initial study. Given the higher density of sigma receptors in the peripheral dorsal root ganglia compared to the dorsal horn or supraspinal brain regions mediating nociception, these results further support the dorsal root ganglia as a target of particular interest for sigma receptor involvement in the various and diverse modalities of pain [35]. Further detailed studies, for instance, selectively eliminating sigma-1 and sigma-2 receptors in nociceptive neurons in cre-lox transgenic mice to evaluate the role of peripheral and central receptors in the nervous system in neuropathic pain states with or without CM-398 treatment, are needed to clarify these remaining points.

Exposure to neuropathic pain is also associated with aversive emotions [36] which diminishes quality of life and complicates therapeutic treatment [37]. We evaluated the effect of CM-398 on the ability to alleviate anhedonia associated with constrictive nerve injury. The affective or emotional component of pain was evaluated with conditioned place preference (CPP), measuring the preference of CCI-exposed mice for the drug-paired compartment of the apparatus [38]. Aversion to pain offers robust motivation to seek pain relief. In this context, associating pain relief with the drug-paired compartment and cues during the occurrence of continued neuropathic pain is reflected by a later conditioned place preference in the absence of the drug [39]. Consistent with this, non-contingent administration of CM-398 in animals exposed to CCI produced conditioned place preference in mice with chronic neuropathic pain, suggesting CM-398 ameliorated chronic discomfort induced by CCI. These results are more remarkable for the fact that the place conditioning of naïve mice with CM-398 was found to produce either no effect or (at 45 mg/kg, i.p.) actual aversion.

Current therapeutics for the treatment of neuropathic pain include gabapentin and morphine, both of which produce significant adverse effects [40,41]. This study sought to evaluate CM-398 for potential liabilities in conditioned place preference, rotarod, and respiratory depression assays. As morphine has a high abuse potential [42], we evaluated CM-398 for liabilities of drug-seeking with the place conditioning assay. At sub-therapeutic and therapeutic doses, CM-398 demonstrated a lack of conditioned place preference (CPP) or conditioned place aversion (CPA), but at high doses (45 mg/kg, i.p.), CM-398 produced CPA consistent with known aversive compounds such as the dopamine-2 receptor agonist, quinpirole [43], or the kappa-opioid receptor agonists U50,488 [44] or U69,593 [45]. These results suggest antagonism of sigma-2 receptors may produce aversion. However, aside from a single demonstration that the non-selective sigma-1 and sigma-2 receptor antagonist AZ-66 produced a conditioned place aversion [9], no studies to date have examined the relationship of sigma-2 receptors and reward responses. While CM-398 was demonstrated to lack affinity for the dopamine-2 receptor or opioid receptors, with binding *K_i_* values greater than 1000 nM in the presence of (−)-[^3^H]sulpiride or [^3^H]naloxone, respectively [17], a detailed examination of the interactions with a full panel of receptors associated with aversion has not yet been completed. It also remains possible that, at the highest therapeutic doses, CM-398 still caused a conditioned place aversion via a non-specific interaction with the kappa-opioid or dopamine-2 receptors. Notably, CM-398 showed no affinity for dopamine-2 or kappa-opioid receptors when tested in a radioligand competition binding assay up to 1 μM concentrations [17]. Further study of CM-398 in competition binding assays for off-target affinity and behavioral testing with sigma-2 receptor and dopamine-2 or kappa-opioid receptor knockout mice would better evaluate these possibilities.

U50,488 also impairs evoked locomotor responses and produces sedative effects [46,47]. At the highest therapeutic doses (45 mg/kg, i.p.), CM-398 produced similar deficits in evoked locomotor activity. Emerging evidence suggests that antagonism of sigma-2 receptors may impair locomotor activity. Consistent with the current results, we previously found that the introduction of sigma-2 receptor antagonism in the non-selective sigma-1 and sigma-2 receptor antagonist, AZ-66, resulted in significant impairment of evoked locomotor activity compared to the sigma-1 receptor-selective antagonist, CM-304 [9]. AZ-66 possesses high affinity for both sigma-2 receptors (0.51 ± 0.15 nM) and sigma-1 receptors (2.4 ± 0.63 nM) but limited selectivity, with a modest sigma-2/sigma-1 receptor selectivity ratio of 4.7 [48]. With high affinity for sigma-2 (0.43 ± 0.015 nM), but not sigma-1 (560 ± 8.73 nM) receptors, CM-398 possesses a far greater sigma-2/sigma-1 receptor selectivity ratio of 1302 [17], correlating with locomotor impairment presently. A potential mechanism detailing how sigma receptor antagonists influence evoked locomotor activity has not yet been elucidated. As more sigma-2 receptor selective ligands are discovered [17,24] and become available for testing, these trends collectively predict that antagonists possessing a higher sigma-2/sigma-1 receptor selectivity may demonstrate sedation.

In evaluation of respiratory effects and spontaneous locomotor activity, CM-398 produced no significant impairment of ambulation at therapeutic doses (30 mg/kg, i.p.) and produced a transient decrease in respiratory rates. These effects may conceivably be attributed to dimerization and the activation of opioid receptors located in the brainstem where sigma-1 receptors are highly concentrated [49]. While outside of the scope of the current studies, future studies might evaluate CM-398 in the presence of an opioid receptor antagonist to test this. Alternatively, the CM-398 might indirectly affect respiration by decreasing locomotor activity, as was ascribed to AZ-66 when it disrupted coordinated locomotion in the rotarod assay [9]. Notably, CM-304 was without significant inhibitory effects on respiration or locomotion, and, in any case, the potential sedative effects of sigma receptor antagonists are poorly understood. Further work is required to assess the effects of the sigma receptors (both sigma-1 and sigma-2) on respiration and locomotor activity, evaluating hypnotic vs. sedative effects.

## 4. Materials and Methods

### 4.1. Materials

#### 4.1.1. Subjects

Adult male C57BL/6J and CD-1 mice, housed five to a cage (8–12 weeks of age), were used. C57BL/6J mice were used in assays of warm-water tail withdrawal [50,51], locomotor and respiration [52], acetic acid writhing [9], and conditioned place preference (CPP) [53,54]. Antinociceptive and anti-allodynic effects were confirmed with CD-1 mice in the formalin inflammatory pain assay and chronic constriction nerve injury assay of neuropathic pain.

All animal studies reported adhere to the ARRIVE guidelines [55]. Animals were randomly assigned, and treatment groups were blinded. Animals were housed on a 12/12 h light/dark cycle (lights off at 7:00 p.m.) with ad libitum access to food and water except during the experimental sessions. All procedures were preapproved by the Institutional Animal Care and Use Committee (University of Florida) and conducted according to the 2011 NIH Guide for the Care and Use of Laboratory Animals.

#### 4.1.2. Materials, Drug Preparation, and Administration

The sigma receptor antagonist CM-304 (FTC146) was synthesized as described previously [56,57], and CM-398 was synthesized as described earlier [17]. All other chemicals and drugs were purchased from Sigma-Aldrich (St. Louis, MO, USA). Sterile saline (0.9%) was used to dissolve U50,488 and morphine. Gabapentin, CM-304, and CM-398 were dissolved in 5% dimethyl sulfoxide (DMSO)/saline. All drugs were administered intraperitoneally (i.p.) in a volume of 250 μL per 25 g of body weight.

### 4.2. Behavioral Assays

#### 4.2.1. Tail-Withdrawal Assay

C57BL/6J mice were used in the 55 °C warm-water tail-withdrawal assay as previously described [52]. Each mouse was independently tested for its initial tail-withdrawal latency prior to drug administration. Latency for tail withdrawal of each mouse was evaluated every 10 min until latency returned to the baseline value post-administration of drug. A maximum response time of 15 s was used to prevent tissue damage, with the failure of the mouse to withdraw the tail within 15 s given a maximum score of 100% antinociception. To account for variability between animals, data are reported as percent antinociception, calculated by the equation:

% antinociception = 100 × ([test latency − baseline latency]/[15 − baseline latency]).(1)

#### 4.2.2. Formalin Assay

The effectiveness of the ligand’s ability to modulate inflammatory pain was performed with the use of C57BL/6J mice in the formalin assay as previously described [9,58]. After a 10 min pretreatment (i.p.) of a graded dose of the vehicle (as a control), morphine (10 mg/kg), or CM-398 (0.01–30 mg/kg), an intraplantar (i.pl.) injection of 5% formalin (2.5 μg in 15 μL) was administered into the right hind paw. Time spent licking the right hind paw was recorded in 5 min intervals for 60 min following injection. The last 55 min of the assessment was used to determine the inflammatory response stimulus. Data were analyzed as the summed duration of licking the hind paw.

#### 4.2.3. Acetic Acid Writhing Test

The ability of CM-398 to modulate chemically induced visceral pain was assessed with the use of C57BL/6J mice in the acetic acid writhing assay as previously described [59,60]. After a 25 min pretreatment (i.p.) of either the vehicle, morphine (1–10 mg/kg), or CM-398 (3–45 mg/kg), a second injection of 0.9% acetic acid (i.p., 0.25 mL per 25 g body wt.) was administered to each mouse. After 5 min, the number of stretches presented in each mouse was counted for 15 min. Antinociception was calculated by the formula:

% antinociception = ([{average stretches in the vehicle group} − {number of stretches in each test mouse}]/[average stretches in vehicle group]) × 100.(2)

#### 4.2.4. Mechanical Allodynia von Frey Assessment

The assessment of mechanical allodynia was performed to measure the anti-allodynic efficacy in the CCI and cisplatin assays as previously described [9,54,57,58,59]. Filaments of increasing pressure (0.4–6 g) were applied to the plantar surface of the hind paw of the mice prior to drug administration to record baseline responses to a peripheral stimulus. Control or test compounds were administered (i.p.), and paw-withdrawal thresholds were again recorded from 20–80 min post-injection. Responses at each time point were measured in triplicate, with clear withdrawal, shaking, or licking of the paw qualified as a response. Data are expressed as percent of baseline paw withdrawal thresholds following stimulation of the ipsilateral hind paw with von Frey filaments. This was utilized to account for innate variability between mice.

% antiallodynia = 100 × ([mean paw withdrawal force {g} in control group − paw withdrawal force {g} of each mouse]/mean paw withdrawal force {g} in control group).(3)

#### 4.2.5. Chronic Constriction Injury

CD-1 mice anesthetized with isoflurane were subjected to chronic constriction injury (CCI), as described previously [9,60] to induce mechanical allodynia and hyperalgesia [58,61,62,63,64]. Briefly, after anesthetization, mice were subjected to surgery where an incision was made along the surface of the biceps femoris of the right hind paw [9]. Blunt forceps were used to split the muscle and expose the right sciatic nerve. The tips of the two 0.1–10 µL pipette tips facing opposite directions were passed under the sciatic nerve to allow for the easy passing of two sutures under the nerve, 1 mm apart. The sutures were tied loosely around the nerve and knotted twice, and the skin was closed with two 9 mm skin staples. The mice were allowed to recover 7 days prior to baseline von Frey testing, as described above, to confirm the induction of mechanical allodynia in each mouse. A response to von Frey fibers of lower force, otherwise not observed in naïve mice, was an indication of mechanical allodynia, consistent with the demonstration of neuropathic pain. The mice confirmed as allodynic were then administered (i.p.) either the controls vehicle (5% DMSO), morphine (10 mg/kg), gabapentin (50 mg/kg), or CM-304 (45 mg/kg), or the test compound CM-398 (10, 30, or 45 mg/kg). Each mouse was then tested for the threshold for mechanical allodynia every 20 min up to 80 min post-treatment with the use of calibrated von Frey filaments as described above, until the threshold that induced paw withdrawal was determined as a measure of nocifensive behavior [9].

#### 4.2.6. Rotarod Assay to Assess Motor Coordination

The rotarod assay of coordinated locomotor response was used to assess the potential sedative effects of the vehicle, U50,488, morphine, or CM-398, as described previously [60]. Seven habituation trials were performed where the last habituation trial was used as an initial baseline of performance. The mice were administered (i.p.) one test agent: vehicle (5% DMSO/95% saline), morphine (10 mg/kg), U50,488 (10 mg/kg), or CM-398 (10, 30 or 45 mg/kg), and then evaluated every 10 min in accelerating speed trials (180 s max latency at 0–20 rpm) for a 60 min period. Latency to fall was measured in seconds. Data are reported as the mean percent change from each mouse’s initial baseline latency to fall. Decreased latencies to fall in the rotarod test indicated impaired motor coordination or sedation.

#### 4.2.7. Respiratory Depression and Spontaneous Locomotor Testing with CLAMS

Spontaneous locomotor activity and respiration rates were measured by the computer automated Comprehensive Lab Animal Monitoring System (CLAMS) (Columbus Instruments, Columbus, OH), as described previously [9]. Unconstrained mice were individually habituated in sealed cages connected to the apparatus for 60 min prior to testing for mouse baseline readings. At the start of testing, mice were intraperitoneally administered drug or vehicle and then placed back into the CLAMS testing cages for 120 min. Respiration rates (breaths/min) were measured using a pressure transducer built into the sealed CLAMS cage. Infrared photobeams located inside each cage measured spontaneous locomotion as the number of photobeam breaks or ambulation. All data are expressed as percent of the vehicle control response.

#### 4.2.8. Conditioned Place Preference

Condition place preference (CPP) was measured with the use of automated, three-compartment place conditioning chambers. C57BL/6J mice underwent a 2-day counterbalanced conditioning design using established methods [9]. Briefly, prior to place conditioning, mice were given free access to all three chambers of the CPP apparatus for 30 min to determine an initial preference. Time spent in each chamber was recorded. Prior to place conditioning, the 106 naïve mice tested did not demonstrate significant differences in the time spent exploring the left (568.2 ± 15.4 s) versus right (573.3 ± 15.7 s) compartments (*p* = 0.86; Student’s t-test). For 2 days after the initial evaluation, the mice were administered the assay vehicle (5% DMSO, i.p.) before being confined in a randomly selected outer compartment of the apparatus for 40 min. After 4 hours, the mice were administered either morphine (10 mg/kg, i.p.), U50,488 (30 mg/kg, i.p.), or CM-398 (10, 30, or 45 mg/kg, i.p.) and then confined to the opposite outer compartment of the apparatus for 40 min. All conditioning parameters were repeated a second day, and 24 h after the second day of conditioning, the final preference was determined by allowing the mice to freely move between the chambers for 30 min. Data are expressed as the difference in time spent in the drug-paired and vehicle-paired compartments. By convention, positive values reflect conditioned preference, whereas negative values are considered conditioned aversion for the drug-paired side.

#### 4.2.9. Chronic Constrictive Nerve Injury/Conditioned Place Preference Operant Model of Pain (CCI/CPP)

To assess the antinociceptive effect of compounds under an operant condition, mice were tested in a procedure modified from Hummel et al. [65] and Salte et al. [66] (see also schematic in Figure 9A). One day prior to CCI surgery, naïve C57BL/6J mice were subjected to initial place preference testing in a three-chambered conditioned place preference apparatus where they were allowed to roam freely for 30 min, as described above. These same mice then underwent chronic constrictive nerve injury and were confirmed after 7 days to demonstrate mechanical allodynia, as detailed above. Allodynic mice were then subjected to 2 days of counterbalanced place conditioning, and the final place preference was assessed, as described above. For place conditioning, mice were treated with vehicle (i.p.) and then randomly confined to one of the outer chambers of the CPP apparatus for 40 min. Four hours later, the mice were administered (i.p.) vehicle, U50,488 (30 mg/kg), or CM-398 (10, 30 or 45 mg/kg) and then confined to the opposite outer compartment of the apparatus. The conditioning was repeated on a second day, and the next day mice were given free access to each compartment of the apparatus for 30 min to determine the final place preference. Data are plotted as the difference in time spent in the drug-paired versus vehicle-paired compartment.

### 4.3. Statistical Analysis

All data are presented as mean ± SEM. Statistical analysis was performed with the use of GraphPad Prism 7.0 software. Significance is indicated as * *p* < 0.05 and was analyzed using Student’s t-test or either one-way or two-way ANOVA with the appropriate post hoc analysis (Dunnett’s, Sidak’s, or Tukey’s) for significant pairwise comparisons within and between groups. Dose response lines were analyzed by linear or nonlinear regression modeling and ED_50_ values (dose yielding 50% effect) along with 95% confidence limits using each individual data point. CLAMS data are reported as the % of matching vehicle control responses, and rotarod data are expressed as the % change from baseline performance, both standard normalizations to compensate for each individual animal’s baseline response. CPP (and CCI-CPP) data are reported as the difference in time spent in the drug and vehicle-paired compartments between preconditioning and postconditioning responses.

## 5. Conclusions

Although limited by uncertainty regarding the mechanistic function of CM-398, the current data suggest that this sigma-2 receptor ligand alleviates inflammatory and chronic neuropathic pain in established mouse models with reduced liabilities. Collectively, these data suggest the therapeutic potential of CM-398 while also contributing further evidence for sigma-2 receptor involvement in analgesic responses.

## Data Availability

The datasets generated for this study are available on request to the corresponding author.
